# The Role of BNP and CRP in Predicting the Development of Atrial Fibrillation in Patients Undergoing Isolated Coronary Artery Bypass Surgery

**DOI:** 10.1155/2013/235018

**Published:** 2013-12-25

**Authors:** Nektarios D. Pilatis, Zacharias-Alexandros Anyfantakis, Kyriakos Spiliopoulos, Dimitrios Degiannis, Antigoni Chaidaroglou, Georgia Vergou, Konstantina Kimpouri, Dennis V. Cokkinos

**Affiliations:** ^1^1st Cardiology Department, Onassis Cardiac Surgery Center, Kallithea, 17674 Athens, Greece; ^2^Department of Cardiology, University of Thessaly, Biopolis, P.O. Box 1400, 41110 Larissa, Greece; ^3^Department of Thoracic and Cardiovascular Surgery, Faculty of Medicine, School of Health Sciences, University of Thessaly, Biopolis, P.O. Box 1400, 41110 Larissa, Greece; ^4^2nd Molecular Immunopathology Laboratory, Onassis Cardiac Surgery Center, Kallithea, 17674 Athens, Greece

## Abstract

*Objective*. To evaluate the association of BNP and CRP with the development of postoperative atrial fibrillation following coronary artery bypass grafting surgery. *Methods*. The series consists of 125 patients (aged 65 ± 9 years), who underwent isolated CABG-surgery. BNP and CRP levels were measured pre- and 24 hours postoperatively and their correlation to the development of postoperative AF was analyzed. *Results*. Forty-four patients (35%) developed AF postoperatively. They were significantly older (68 ± 8 versus 63 ± 9, *P* = 0.01) and predominantly nonsmokers (18% versus 46%, *P* = 0.004), compared to the non-AF cases. In addition they showed significant higher preoperative mean BNP levels of 629 versus 373 pg/mL (*P* = 0.019). Postoperative BNP levels were significantly higher in both groups (AF-group: 1032 pg/mL versus non-AF group: 705 pg/mL; *P* < 0.001), while there was a trend of more increased postoperative levels in AF-cases (*P* = 0.065). AF-episodes appeared significantly more frequent in the two highest quartiles of BNP levels with 44% (*P* = 0.035). On the contrary pre- and postoperative CRP levels were not associated with AF. Multivariable analysis revealed only increased preoperative BNP levels as independent predictor for postoperative AF (*P* = 0.036). Conclusion. Elevated preoperative BNP serum levels are associated with the development of post-CABG AF, while CRP does not seem to be influential.

## 1. Introduction

Atrial fibrillation (AF) occurs in 25–40%, representing the most common arrhythmia in patients undergoing isolated coronary artery bypass surgery (CABG) [1–3]. Although it is not a life-threatening rhythm disturbance and may present as self-limiting onset, it has major medical and economical implications. It may compromise cardiac function, increase 2- to 3-fold the risk of stroke and thromboembolism, result in iatrogenic complications due to additional treatment efforts, prolong hospitalization duration (by 1–3 days), and elevate treatment cost [3–7].

Numerous studies, mainly retrospective, have been conducted to clarify the pathogenesis of postoperative AF as well to identify predisposing factors. However, the exact etiologic pattern still remains unclear. The proposed contributory factors include inflammation triggered by cardiopulmonary bypass, beta-blocker withdrawal, right coronary artery stenosis, atrial ischemia, inadequate intraoperative cardiac protection, perioperative ischemic injury, postoperative pericarditis, autonomic imbalance, and fluid/electrolyte disturbances during the intra- and postoperative periods [8–14].

Brain natriuretic peptide (BNP) is released in circulation mainly from left atrial and ventricular myocytes due to pressure and/or volume overload in the heart chambers [15]. The hormone promotes natriuresis, diuresis, and vasodilatation, while blood levels of BNP are raised in patients with structural cardiac disease, particularly those with heart failure.

High-sensitivity C-reactive protein (hs-CRP) is an acute-phase protein and an established marker of inflammation. In addition elevated CRP levels have been linked to the severity of atherosclerosis, risk of coronary events and even long-term outcome after CABG [16]. Its arrhythmogenic effect has been postulated to be related to sodium and calcium exchange disturbances following its linkage to phosphocholine [17].

All the aforementioned clinical entities offer a suitable milieu for the development of AF and as long as both biomarkers are related with those onsets; we investigate in our series the role of BNP and CRP in predicting the occurrence of post-CABG AF.

## 2. Patients and Methods

In this prospective, single center study 125 consecutive patients undergoing isolated coronary artery bypass graft operation (CABG), over a 6-month time period at the Onassis Cardiac Surgery Center were enrolled.

Indications for CABG surgery were significant (>60%) disease of the left main coronary artery stem, ostial stenosis of left anterior descending artery, two- or three-coronary-vessel disease, and symptomatic coronary artery disease unsuitable for percutaneous coronary intervention. All cases were operated on an elective basis. The decision to proceed with “on-” or “off-pump” operation was based upon the surgeons' preference, which was mainly influenced by patients/disease characteristics. However, there were no uniform criteria among the surgeons for the use of either technique.

Patients considered to be at high risk for the development of postoperative AF, like those with history of AF on antiarrhythmic medical therapy, congestive heart failure at the time of preoperative evaluation, and/or concomitant valve surgery, those suffering from a chronic inflammatory condition, and/or those under medical treatment with amiodarone, corticosteroids, or non-steroidal antiinflammatory drugs within 30 days prior to CABG surgery, were excluded from the study.

In all cases the left ventricular function was evaluated preoperatively by transthoracic echocardiogram. The extent of diseased coronary vessels as well as the number of distal coronary anastomoses performed on each case was recorded. Patients undergoing “on-pump” surgery received either antegrade or retrograde cardioplegia. The decision to use intra-aortic balloon pump, cardiac inotropic support, or temporary pacing was made by the anesthesiologist and/or the surgeon and was determined by the patient's hemodynamic status and rhythm in the operating room and the postoperative heart surgical unit. Efforts were made to extubate patients within 24 hours after surgery. All patients were followed on telemetry for at least five days postoperatively or until discharge from the hospital, achieving in that way in all cases continuous rhythm monitoring. Atrial fibrillation was defined as any episode of the arrhythmia lasting longer than one hour and requiring treatment.

Blood samples were collected from all patients the day before surgery and 12 to 24 hours after the operation, on the first postoperative day for hemoglobin concentration and routine chemistry values.

### 2.1. BNP Measurement

Samples for BNP determinations were collected after patients had remained in supine position for 30 minutes. Blood samples were centrifuged within 30 minutes, at 3000 c/min for 10 minutes at 4°C. Plasma was extracted and stored at −70°C until further analysis. For the measurement of BNP plasma concentrations, a competitive enzyme immunoassay kit from Biomedica (Wien, Austria) that utilizes a polyclonal BNP 8–29 fragment antibody directed at the BNP molecule was used. Inter-assay and intraassay variabilities were 3.8 to 4.4% and 4.0 to 6.5%, respectively.

### 2.2. CRP Measurement

Sera samples were collected in BD SST gel clot vacutainer tubes (Becton Dickinson, San Jose, USA) and centrifuged within 30 min of venipuncture at 3500 rpm for 10 min and stored at −20°C until measurement. The FDA approved Cardio-phase hs-CRP particle enhanced immunonephelometric assay was used in a BN Prospec nephelometer (Date Behring Siemens, Marburg, Germany). The assay was calibrated against WHO ERM-DA470 reference material.

The study population was divided in two groups according to the postoperative development of arrhythmia (AF and non-AF). They were compared for differences in baseline demographic, clinical, and operative characteristics and the measured levels of serum BNP and inflammatory marker CRP. Subgroup analysis was performed for “off-” versus “on-pump” operated cases. The study was approved by the institution's ethics committee and all patients participating in the study signed an informed consent.

## 3. Statistical Analysis

Data analysis used software from the statistical package of “SPSS for Windows, Release 13.0” (SPSS Inc., Chicago, IL, USA). All categorical values appeared in the series are presented as percentages (values were rounded to the closest unit-number), while quantitative variables are given as means with 95% confidence intervals (95% CI), unless otherwise noted. All numerical variables were tested with the Kolmogorov-Smirnov and Shapiro-Wilk tests for normality of their values' distribution. Groups of AF- and non-AF patients were compared by (a) chi-square test or Fisher's exact test for their baseline clinical and operative characteristics and (b) independent-sample *t*-test (or Mann-Whitney *U* test), whichever was appropriate, for age, BNP, and CRP.

Subsequently, the study collective was divided into two subgroups, including patients with BNP or CRP levels either above or below the median value and comparisons with regard to the postoperative AF incidence were performed. The same comparison was performed between patients in the low and high quartile of the above mentioned markers.

Correlation between age, BNP, and CRP was tested using Pearson and Spearman's correlation coefficients.

To clarify the role of BNP or CRP in predicting the development of postoperative AF, multivariable logistic regression analysis was performed, adjusted for gender, arterial hypertension (HTN), off-pump operation, postoperative beta-blocker administration, and the predictors of atrial fibrillation, delivered by univariate analysis. In case of a positive correlation between two numerical predictors, only one was entered in the calculations. Hosmer and Leme show goodness-of-fit test confirmed the validity of the model (the assumptions of the model were met) in all cases of multivariate prediction of postoperative AF. Level of significance for *P* value was set at 0.05.

## 4. Results

From the patients operated by two surgical teams participating in the study, 125 (mean aged 65 years, SD = 9, 118 males) fulfilled the inclusion criteria. Regarding their demographic characteristics two-thirds of them were hypertensives and one third diabetics. There was a high incidence of beta-blocker (79%) and ACEI (62%) preoperative use and only 26% were treated with calcium channel blockers. Surgery was performed without the use of cardiopulmonary bypass (“off-pump”) in 18% of the cases. Postoperatively the majority (75%) was extubated within 24 hours after surgery, while 78% received for analgesia acetaminophen and codeine preparation.

Forty-four individuals (35%) developed AF in the postoperative phase during days 1 to 5 (mean = 3), which lasted up to 6 days (median = 2 days). Most of the patients (71%) converted to sinus rhythm after intravenous administration of amiodarone.

The comparison of AF- with non-AF patients, depicted in Table 1, showed that AF patients were significantly older (*P* = 0.01), smoked less (*P* = 0.004), and had a longer hospital stay (*P* = 0.03) than their non-AF counterparts. All other evaluated baseline characteristics were similar in both groups.

**Figure 1:**
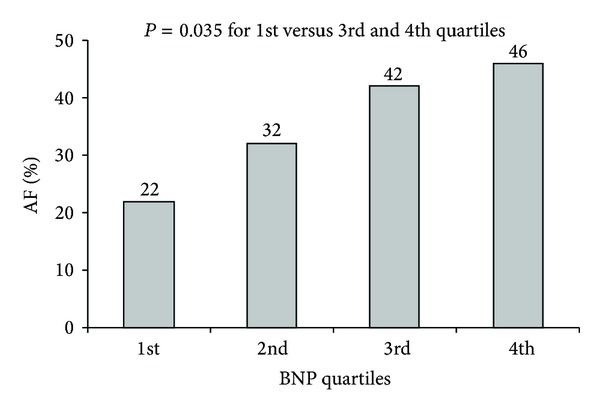
Incidence of postoperative AF by preoperative BNP quartiles.

Concerning preoperative BNP levels, they were significantly higher in patients who subsequently developed postoperative AF with a mean value of 629 versus 373 pg/mL (*P* = 0.019) in the non-AF group (Table 2). Postoperative BNP-levels were also significantly higher than the preoperative ones in both groups (AF-group: 1032 pg/mL versus non-AF group: 705 pg/mL; *P* < 0.001), while there was a strong trend of more increased postoperative levels in AF- compared to the non-AF cases (*P* = 0.065). Patients in the highest two quartiles of BNP developed AF significant more frequently than those in the lowest quartile (46% and 44% versus 22%, *P* = 0.044 and *P* = 0.035) (Figure 1). Similar results led to a dichotomization of the study collective in two subgroups according to the preoperative BNP concentration using as “cut-off” point the median value. Patients of the high BNP subgroup (above the median value) demonstrated a marginal nonsignificant higher incidence than the low subgroup of presenting postoperatively AF (43% versus 27%, *P* = 0.071).

The same calculations were performed evaluating the role of CRP. The analysis revealed that there was no difference between pre- and postoperative levels of CRP among AF- and non-AF patients. Additionally no significant difference was observed in the incidence of postoperative AF by quartiles of CRP levels (Table 2).

Correlation analysis of the entire series population demonstrated a positive interaction between pre- and postoperative BNP levels with age (*r* = 0.25 and 0.35; *P* = 0.014 and 0.004, resp.) as well as between the pre- with postoperative BNP levels (*r* = 0.65 and *P* < 0.001).

Further subgroup analysis, comparing “off-“ with “on-pump” operated patients, showed that non-AF, “off-pump” operated cases were accompanied by significant (*P* = 0.015) much lower postoperative BNP levels than their “on-pump” counterparts, despite their similar preoperative BNP values. On the other hand, in the AF-cases there were no significant differences between BNP levels, regardless of preferred surgical technique (Table 3).

A subsequently performed multivariable regression analysis searching for independent predictors of postoperative AF identified only increased preoperative BNP levels (being in the two highest quartiles of BNP) as predictor of this arrhythmia after adjustment for age (above or below the median value), CRP levels, postoperative beta-blockers administration, gender, HTN, smoking, and “off-pump” operation, with odds ratio (OR) of 0.56 (95% CI = 0.32–0.96; *P* = 0.036). On the other hand CRP did not emerge, as an independent predictor for the complication.

## 5. Discussion

Risk stratification of patients undergoing cardiac surgery remains challenging. Several methods, including clinical risk scores (i.e., EuroSORE, STS score, Parsonnet score) as well as measurement of biomarkers (BNP/NT-pro-BNP, cTn, CRP) have been proposed to assess the patient's risk for adverse outcomes after cardiac operations. While on the one hand scoring systems are limited by their suboptimal performance in predicting major postoperative morbidity, as well as the potential for deterioration in discrimination and calibration over time [18], on the other hand monitoring of biomarkers, although widely used for cardiovascular evaluation in emergency departments, is lacking of evidence regarding their utility in stratifying cardiac surgical populations. Aim of our series is to address this issue by investigating possible correlations of BNP and CRP to postoperatively developed AF in patients after isolated CABG. Its results apply to isolated CABG patients and all data were prospectively collected, meaning that investigators were blinded to BNP and CRP levels at the time of AF status assessment.

The post-CABG AF incidence of 35% in our study concurs with that reported by numerous groups [1–3]. Main finding of performed analyses is that elevated preoperative BNP level in patients undergoing isolated CABG is a strong independent predictor of postoperative AF development. Although univariate analysis identified additionally age as significant predictor, this finding was not confirmed by multivariate analysis, where only serum BNP remained predictive. In the literature more studies support [17, 19, 20] than refute [21] this observation.

Regarding the postoperative BNP levels, although we demonstrated a strong trend to AF emergence, this correlation did not reach statistical significance, as it did in a study by Song et al. [22].

The finding of significant lower immediate postoperative BNP levels in non-AF “off-pump” cases delivered by the performed subgroup analysis comparing “on-“ with “off-pump” operated patients underscore the triggering effect, which may have cardioplegia, the cardiopulmonary bypass per se, and in a lesser extent manipulations of the heart during the “cardiac arrest” phase on the intraoperative release of BNP. In contrast Mahar and coworkers showed in their series that peak BNP values appear to be no different between those undergoing CABG surgery with or without cardiopulmonary bypass [23], while Guerin et al. reported that the time to peak BNP value appears to be slightly slower for those undergoing cardiopulmonary bypass [24]. These discrepancies to other researchers' results may be attributed on the one hand to the relative small sample size of 18% in our cohort undergoing “off-pump” surgery, and on the other hand on the timing of postoperative BNP measurement (early on 1st POD) “missing” in that way some of the peak values. Apart from that, both above mentioned studies confirmed the predictive power of preoperative BNP level for postoperative AF irrespective of surgical technique used.

Various treatment protocols including administration of digitalis, ACE inhibitors, and calcium channel- and beta-blockers, have been conducted to prevent or treat post-CABG AF [10, 25]. ACE inhibitors and beta-blockers but no amiodarone were used quite frequent preoperatively in our patients, yet they were not significant influential in our evaluation.

Our series presented the expected rise in CRP associated with CABG surgery, but there was no variability between the biomarker and post-CABG AF, a finding widely documented in the literature [17, 26, 27]. Gasparovic et al. in their recently published study including 215 only “on-pump” cases clearly demonstrated, by incorporating in their calculations in contrast to other researchers CRP measurements at three different time points, that the magnitude of the inflammatory response does not influence the incidence of postoperative AF [17]. Thus, the inflammation marker CRP cannot be postulated to be predictive for post-CABG AF.

Our results showed that BNP but not CRP concentrations provide predictive information, on the risk of developing AF after isolated CABG surgery, which is not captured by standard clinical scoring systems such as EuroSCORE. Apart from the study of Cuthbertson et al., reporting an additive value of preoperative BNP levels and risk with an adjusted 3% increase in risk of death for each 250 pg/mL rise postoperatively [28], the total number of studies examining BNP is limited; thus cut-off points for patient's risk stratification have yet to be identified. However preoperative measurements of BNP may be useful in stratifying patients into different risk categories, enabling prophylactic interventions including pretreatment of high risk patients with amiodarone or other forthcoming still under investigation antiarrhythmic agents. In general evaluation of preoperative natriuretic peptides levels contribute to the individual surgeon-patient discussion and might also aid the planning of surgical lists, making in that way the use of resources more efficient.

On the other hand, improvement in the performance and generalizability of many existing clinical prognostic models may be achieved not only by recalibration and readjustment but also through the incorporation of biomarkers measurements like BNP/NT-pro-BNP. This should be certainly further tested and evaluated in prospective randomized controlled trials.

### 5.1. Limitations of the Study

There are certain limitations which warrant mentioning.

Firstly this prospective series refers to a single center regional experience, thus the results may not be generalized for the entire population, since there are significant differences between institutions and countries.

Secondly the total sample size of 125 patients with a rate of only 18%, as noted before, of “off-pump” operated cases is relatively small to avoid biases in our evaluation.

Finally continuous rhythm monitoring took place for the first five postoperative days and in some cases till the discharge from the hospital. Any paroxysmal AF episode occurring afterwards is not recorded and therefore not evaluated.

## 6. Conclusions

Despite the above mentioned limitations, our study revealed some trends, which in accordance to other series' results allow us to draw following conclusions.

Elevated preoperative serum levels of BNP are definitely associated with the development of post-CABG AF, while this was not the case for the inflammation marker CRP. Preoperative measurements of natriuretic peptides should be considered in the conduction of risk stratification algorithms in order to identify potential patients who will develop AF after CABG surgery.

## Figures and Tables

**Table 1:** Patient characteristics by postoperative AF.

	AF (*n* = 44)	Non-AF (*n* = 81)	*P* value
Age (y) (mean ± SD)	68 ± 8	63 ± 9	0.01
Sex (male) [*n* (%)]	43 (98)	75 (93)	ns
Art. HTN [*n* (%)]	33 (75)	50 (62)	ns
DM [*n* (%)]	15 (34)	23 (28)	ns
Renal insufficiency [*n* (%)]	1 (2.3)	2 (2.5)	ns
Nicotin abuse [*n* (%)]	8 (18)	37 (46)	0.004
Alcohol abuse [*n* (%)]	1 (2.3)	2 (2.5)	ns
ACEI preop. [*n* (%)]	29 (66)	48 (59)	ns
Diuretics preop. [*n* (%)]	11 (25)	15 (18.5)	ns
Digitalis preop. [*n* (%)]	2 (4.5)	2 (2.5)	ns
CCB preop. [*n* (%)]	15 (34)	18 (22)	ns
Beta-blocker preop. [*n* (%)]	32 (72.8)	66 (81.5)	ns
LIMA [*n* (%)]	41 (93)	80 (99)	ns
SVGs ≥ two [*n* (%)]	27 (61.5)	41 (51)	ns
SVG →RCA [*n* (%)]	27 (61.5)	39 (48)	ns
“Off-pump” surgery [*n* (%)]	4 (9)	18 (22)	ns
Extubation within 24 hrs [*n* (%)]	32 (72.8)	63 (77.7)	ns
Hospital stay (d) (median)	7	6	0.03
Hemoglobin (g/dL) (mean ± SD)	14.0 ± 1.2	13.8 ± 1.5	ns
Blood urea (mg/dL) (mean ± SD)	40 ± 17	46 ± 22	ns
Serum Creatinine (mg/dL) (mean ± SD)	1.0 ± 0.2	1.1 ± 0.3	ns
Na^+^preop. (mmol/mL) (mean ± SD)	140 ± 2	140 ± 3	ns
K^+^preop. (mmol/mL) (mean ± SD)	4.5 ± 0.4	4.6 ± 0.7	ns

AF: atrial fibrillation; *n*: number of cases; y: years; SD: standard deviation; Art. HTN: arterial hypertension; DM: diabetes mellitus; ACEI: angiotensin converting enzyme inhibitors; CCB: calcium channel blockers; preop: preoperative; LIMA: left internal mammary artery; SVG: saphenous vein graft; RCA: right coronary artery; hrs: hours; ns: nonsignificant.

**Table 2:** CRP and BNP levels in pg/mL (mean, 95% CI) by postoperative AF status.

Biomarker (pg/mL)	AF (*n* = 44)	Non-AF (*n* = 81)	*P* value
CRP preop.	4.8 (1.9–7.8)	3.95 (2.4–5.5)	0.43
CRP postop.	114.56 (93–137)	128 (105–152)	0.94

BNP preop.	629 (334–924)	373 (312–435)	**0.019**
BNP postop.	1032 (1094–1369)	705 (588–823)	0.065

CRP: C-reactive protein; BNP: brain natriuretic peptide; preop: preoperative; postop: postoperative; other abbreviations are like in Table 1.

**Table 3:** BNP levels (pg/mL) in “off-” versus “on-pump” operated patients with regard to postoperative AF.

	Surgical Technique					
	“Off-pump” (*n* = 23)			“On-pump” (*n* = 102)		
Group	Mean	Median	SD	Mean	Median	SD
AF						
BNP preop.	401.94	410.63	129.22	466.87	427.00	366.93
BNP postop.	698.15	656.44	372.65	757.70	613.30	525.60
Non-AF						
BNP preop.	315.10	300.60	151.53	430.26	320.50	316.76
BNP postop.	475.35	411.93*	161.57	860.03	740.34*	539.76

Non-AF “on-pump” operated patients had significant higher postoperative BNP levels than “off-pump” patients (*P = 0.015).
